# Deep learning model utilizing clinical data alone outperforms image-based model for hernia recurrence following abdominal wall reconstruction with long-term follow up

**DOI:** 10.1007/s00464-024-10980-y

**Published:** 2024-06-11

**Authors:** Hadley H. Wilson, Chiyu Ma, Dau Ku, Gregory T. Scarola, Vedra A. Augenstein, Paul D. Colavita, B. Todd Heniford

**Affiliations:** 1 Division of Gastrointestinal and Minimally Invasive Surgery, Department of Surgery, Carolinas Medical Center, 1025 Morehead Medical Drive Suite 300, Charlotte, NC 28204 USA; 2 Department of Statistical Science, Duke University, Durham, NC USA

**Keywords:** Artificial intelligence, Deep learning, Ventral hernia, Abdominal wall reconstruction, Hernia recurrence

## Abstract

**Background:**

Deep learning models (DLMs) using preoperative computed tomography (CT) imaging have shown promise in predicting outcomes following abdominal wall reconstruction (AWR), including component separation, wound complications, and pulmonary failure. This study aimed to apply these methods in predicting hernia recurrence and to evaluate if incorporating additional clinical data would improve the DLM’s predictive ability.

**Methods:**

Patients were identified from a prospectively maintained single-institution database. Those who underwent AWR with available preoperative CTs were included, and those with < 18 months of follow up were excluded. Patients were separated into a training (80%) set and a testing (20%) set. A DLM was trained on the images only, and another DLM was trained on demographics only: age, sex, BMI, diabetes, and history of tobacco use. A mixed-value DLM incorporated data from both. The DLMs were evaluated by the area under the curve (AUC) in predicting recurrence.

**Results:**

The models evaluated data from 190 AWR patients with a 14.7% recurrence rate after an average follow up of more than 7 years (mean ± SD: 86 ± 39 months; median [Q1, Q3]: 85.4 [56.1, 113.1]). Patients had a mean age of 57.5 ± 12.3 years and were majority (65.8%) female with a BMI of 34.2 ± 7.9 kg/m^2^. There were 28.9% with diabetes and 16.8% with a history of tobacco use. The AUCs for the imaging DLM, clinical DLM, and combined DLM were 0.500, 0.667, and 0.604, respectively.

**Conclusions:**

The clinical-only DLM outperformed both the image-only DLM and the mixed-value DLM in predicting recurrence. While all three models were poorly predictive of recurrence, the clinical-only DLM was the most predictive. These findings may indicate that imaging characteristics are not as useful for predicting recurrence as they have been for other AWR outcomes. Further research should focus on understanding the imaging characteristics that are identified by these DLMs and expanding the demographic information incorporated in the clinical-only DLM to further enhance the predictive ability of this model.

Hernia recurrence has been the traditional benchmark of success for elective hernia repair. A recurrence negatively impacts quality of life postoperatively, causes patients to question whether their surgery was necessary or adequate, and leaves the need for an additional operation [1, 2]. Patients presenting with a recurrence tend to be more complex, leading to a greater chance of complications and further recurrence when an additional operation is performed [3, 4]. Therefore, a recurrence after abdominal wall reconstruction (AWR) can lead to a “vicious cycle” of complications and further recurrences [5, 6]. These complications are burdensome to the healthcare system. It has been estimated that over 500,000 ventral hernia repairs (VHRs) are now being performed in the United States annually, representing health care costs exceeding $3 billion. Of those total numbers, patients with a recurrence have been shown to account for more than 20% of those undergoing incisional hernia repair [7, 8]. Of late, research in hernia surgery has shifted to quality of life and other important metrics. But, given the frequency, cost and psychosocial impact of a failed hernia surgery, many randomized controlled trials in the field continue to focus on hernia recurrence as their primary outcome [9–13].

Predicting patients who are at increased risk for hernia recurrence preoperatively may help to guide management strategy and improve shared decision-making with AWR patients. For example, greater emphasis may be placed on preoptimization – usually centered around smoking cessation, glycemic control, and weight loss – in high-risk patients prior to surgery, or nonoperative management may be considered in those at especially high risk [9]. Additionally, these factors might suggest referral to a tertiary AWR surgeon and facility. A recent meta-analysis identified 22 different predictors of recurrence [14]. Synthesizing this amount of patient information and translating it into a meaningful risk calculation is a daunting task for a preoperative clinic visit. Various risk stratification tools for AWR have thus been developed in an attempt to streamline this process. Unfortunately, many of these tools have considerable limitations as they do not assess the risk of recurrence specifically, are still overly cumbersome, or lack external validity [15]. There remains a need for a predictive tool for recurrence available to AWR surgeons.

Deep learning has the ability to efficiently analyze complex patient data and generate an accurate prediction of surgical outcomes. Briefly, deep learning is a subcategory of artificial intelligence (AI), a field of computer science in which computer systems mimic human cognitive function [16]. The authors have previously reported on a deep learning model (DLM) that was able to predict the need for component separation in AWR, outperforming a panel of expert AWR surgeons in the same task [17]. DLMs have also been shown to accurately predict postoperative outcomes including surgical site infection, mesh infection, and pulmonary failure [17, 18]. Interestingly, these DLMs have been able to make these predictions based solely on the patients’ preoperative computed tomography (CT) images. Recently, Hassan et al. also reported on the use of AI to predict recurrence, complications, and 30-day readmission following AWR. Their model did not incorporate preoperative imaging, but rather a number of clinical variables, to make predictions [19]. Given the previous success of CT image-based DLMs to predict surgical outcomes after AWR, the goal of this study was to develop a model that could predict hernia recurrence. The authors further hypothesized that incorporating clinical data into the DLM would enhance the predictive ability of the image-based model.

## Materials and methods

### Study population and design

Institutional review board approval was obtained prior to conducting this study. Patients were identified from a prospectively maintained database at a tertiary hernia referral center. Patients were included who underwent AWR and had preoperative CT imaging of their hernia available. The CT images had to be within 1 year prior to their operation to meet the inclusion criteria, and only images containing the hernia defect were included. Patients were excluded if there were missing images or if there was significant distortion of their CT images (for example, from an orthopedic prosthesis). Other exclusion criteria included age < 18 years old, undergoing an emergent operation, or having follow up < 18 months. This follow-up cutoff was based on previous data showing that < 50% of incisional hernia recurrences are captured within 1 year of follow up, but a majority are captured after 1–2 years of follow up [8]. Preoperative and operative characteristics and postoperative recurrence data were collected. Hernia defect size was calculated as a surface area as width x length based on measurements reported in the operating surgeon’s operative note. Recurrence was determined by physical exam documented by a provider.

Preoperative CT images were deidentified and prepared using the TeraRecon software (TeraRecon, Inc., Durham, NC). Axial slices of the abdomen that contained the hernia were included. This methodology meant that some patients would have a greater number of image slices, depending on the hernia defect size. The slices were 3–5 mm in thickness and images were standardized to 150 × 150 pixels.

All operations were performed by specialty-trained AWR surgeons at a single high-volume center. AWR refers to the practice of performing hernia repairs with the goal of restoring the structure and function of the abdominal wall [20]. The practice of these surgeons in terms of preoperative optimization and operative technique is similar and has been described previously [21]. Patients who are smoking are required to quit at least 4 weeks prior to surgery, and a preoperative urine cotinine test is used to confirm adherence to this requirement. An A1c of 7.2 or less is targeted for patients with diabetes. Appropriate counseling and referral are provided to assist patients in meeting these goals. There is not a strict cutoff for body mass index (BMI), but generally patients with BMI > 35 kg/m^2^ are counseled to lose weight before surgery is performed. Instruction in a ketogenic diet is provided, and exercise is encouraged. Once patients are optimized for surgery, AWR is usually performed with an open preperitoneal approach as was done in the vast majority of patients in this study. Patients are given preoperative antibiotic and venous thromboembolism prophylaxis, and for an open operation a midline incision is performed. The hernia contents are reduced, and lysis of adhesions is performed as necessary to remove any adhesions to the anterior abdominal wall. Whenever possible, thorough dissection of the preperitoneal space is then accomplished to allow for placement of a large mesh into this space with a wide overlap of the mesh beyond the hernia defect 5–10 cm in all directions. Generally, a midweight polypropylene mesh is used in clean and clean-contaminated cases unless the patient is at higher risk for developing or not being able to tolerate a mesh-related complication, such as transplant or immunocompromised patients [22]. In contaminated and dirty cases, we preferentially use biologic mesh as our data suggests less mesh-related complications in these settings [23]. The mesh is secured with transfascial suture fixation to the anterior abdominal wall. The peritoneum is closed prior to mesh placement, and the fascia is closed over the mesh. It is the goal to achieve fascial closure when possible, and a component separation, either a transversus abdominis release or an external oblique release, is performed when necessary. To assess for tension and the need for a component separation, Kocher clamps are placed on the anterior fascia and pulled together. If the closure is felt to be on tension, the posterior rectus sheath is first released. If there is still felt to be tension on the fascial closure and the space needed for the fascia to come together is 6 cm or less, a transversus abdominis release is performed. If there is need for a greater release, then an external oblique release is performed with an effort to spare the periumbilical perforator vessels. The midline incision is typically closed with absorbable deep dermal sutures, staples, and an incisional negative pressure dressing. Of note, there were a small subset of patients in this study who had their surgery performed laparoscopically due to surgeon preference. An intraperitoneal underlay mesh was placed in these cases. One patient had significant intraabdominal adhesions requiring a conversion to an open incision for completion of lysis of adhesions. In this specific case, an intraperitoneal underlay mesh was placed laparoscopically after the fascia was closed.

### Model development

A trained computer scientist developed the initial DLM based on preoperative CT images and a binary outcome (yes/no) of recurrence following AWR. Another model was developed to train on basic patient data: age, sex, history of tobacco use, history of diabetes, and BMI. The two models were then integrated to create the mixed-value model. Our prior experience with blending imaging and objective data has led to overfitting, or overlearning, of DLMs so that they are unable to consider further variations of information [18]. Thus, there is a fine line where including too many variables may render a model impractical, so the rationale was to use limited and relatively basic clinical variables, readily available for almost any patient, to build the model. Patients were randomized into a training set (80%) and a testing set (20%). The DLMs were blinded to the test set until internal validation was performed. The DLMs used an Adam optimizer with a learning rate of 0.1 and binary cross entropy loss.

In previous image-based DLMs for AWR outcomes, every image slice containing the hernia defect has been used as an input [17, 18]. Initially, the same strategy was used here, but there was a substantial amount of noise introduced by using every image, making it impossible to construct a reliable model. In collaboration with our data scientists, we determined that not every slice would capture the representative features of a hernia. In an effort to reduce irrelevant information contained in the images and focus on the relevant hernia characteristics, another strategy was used in this study by instead using a frame averaging technique, accomplished by the following algorithm: Output = { $\frac{1}{\text{N}}*{\sum }_{n=1}^{N}pixel\left(\text{n},\text{x},\text{y}\right)$|all pixels in the image set} with x and y representing the x, y coordinates of the pixel in a given image, and n representing the image number within the set. A single averaged image for each patient was produced for the DLMs to predict recurrence.

The image-only DLM was designed as a convolutional neural network (CNN) with two convolutional layers and one linear layer that trained with the averaged images. The image data was passed through the two convolutional layers with a window size of 5 × 5. The output embedding from the convolutional layers was passed to a linear layer and a dropout layer with a probability of 0.3 to train a node within the layer. The final output was passed to a sigmoid activation function with a logit of ≥ 0.5 indicating a prediction of recurrence and < 0.5 predicting no recurrence.

The clinical-only DLM was designed as a five-layer feedforward neural network (FNN) that trained on the clinical data alone. The patient characteristics passed through two batch norm layers followed by a dropout layer with a probability of 0.2. A logit was similarly returned predicting whether a recurrence would occur.

Finally, the mixed-value DLM was designed as another FNN, incorporating the logits returned by the image-only and the clinical-only DLMs. The logits were concatenated by an interpolation network and fit into two linear layers, producing a single logit used to determine the prediction of recurrence.

### Statistical analysis

Statistical analyses were performed by a trained statistician using the Python Software Foundation (Python Language Reference, version 2.7) and SAS program version 9.4 (SAS, Cary, NC, USA). Categorical variables were reported as frequencies and percentages. For categorical variables with missing data, the patients with missing data were considered a “no” for the purposes of reporting summary statistics so that the reported frequencies/percentages were only those that were confirmed to be a “yes.” Continuous variables were reported as the mean ± standard deviation. Comparisons of preoperative and operative characteristics were performed using the Kruskal–Wallis test or Student’s t-test for continuous variables and the Pearson χ^2^ or Fisher’s exact test for categorical variables, where appropriate. A two-tailed statistical significance was set at p < 0.05 before data collection. The primary outcome was the ability of the DLMs to differentiate between patients who did or did not have a hernia recurrence over the specified follow up period by the area under the curve (AUC) of the receiver operating characteristic (ROC) curve. As previously described, an AUC ≥ 0.7 was considered the threshold to be considered a predictive model [24].

## Results

There were 190 patients included in this study. Preoperative and operative characteristics are reported in Table 1. Of the patient characteristics included in the models, there were 125 (65.8%) females, and patients had an average age of 57.5 ± 12.3 years old. Patients had an average BMI of 34.2 ± 7.9 kg/m^2^, and there were 53 (27.9%) with a history of diabetes and 35 (18.4%) with a history of tobacco use. A majority of patients (54.7%) presented with a recurrent hernia, and the average number of previous hernia surgeries was 2.1 ± 1.5. These were on average very large hernias with a defect size of 177.5 ± 183.7 cm^2^. Mesh was placed in 93.2%, and 95.8% had an open procedure performed. The fascial defect was completely closed in 91.1% (1.1% of patients missing data), and a component separation was required in 26.3% (2.6% of patients missing data). Postoperatively, 28 (14.7%) experienced a recurrence with a median follow up of more than 7 years (85.4 [56.1, 113.1] months). For the most part, patients who had a recurrence did not have statistically significant differences in their preoperative and operative characteristics from those who did not (Table 1). The exception was that there was a statistically significant higher proportion of patients repaired laparoscopically in the group with recurrences (14.3% vs 1.9%, p = 0.005). There were no statistically significant differences in characteristics for the patients in the training set and testing sets (Table 1). Of the 28 total patients with a recurrence, there were 22 (14.5%) in the training set and 6 (15.8%) in the testing set (*p* = 0.838).

**Table 1:** Preoperative and operative characteristics

	Overall	No recurrence	Recurrence	*p*-value	Testing	Training	*p*-value
	*N* = 190	*N* = 162	*N* = 28		*N* = 38	*N* = 152	
Age (Years)	57.5 ± 12.3	57.4 ± 12.3	58 ± 12.4	0.757	58.8 ± 12.0	57.2 ± 12.4	0.485
Sex (Female)	125 (65.8)	108 (66.7)	17 (60.7)	0.540	24 (63.2)	101 (66.5)	0.702
BMI (kg/m^2^)	34.2 ± 7.9	33.7 ± 7.5	37.2 ± 9.4	0.086	34.8 ± 8.3	34.1 ± 7.8	0.671
History of diabetes	53 (27.9)	45 (27.8)	8 (28.6)	0.931	9 (23.7)	44 (29.0)	0.518
History of tobacco use	35 (18.4)	28 (17.3)	7 (25.0)	0.331	5 (13.2)	30 (19.7)	0.349
Number of comorbidities	4.6 ± 2.6	4.5 ± 2.7	4.7 ± 2.3	0.616	5.2 ± 2.8	4.4 ± 2.6	0.129
Recurrent hernia	104 (54.7)	86 (53.1)	18 (64.3)	0.272	20 (52.6)	84 (55.3)	0.771
Previous hernia surgeries	2.1 ± 1.5	2.1 ± 1.4	2.4 ± 2.1	0.620	2.5 ± 2.1	2.0 ± 1.4	0.655
Hernia defect size (cm^2^)	177.5 ± 183.7	183.7 ± 190.2	134.8 ± 126.7	0.328	212.1 ± 196.6	168.5 ± 180.0	0.225
*Mesh type*				0.457			0.435
Synthetic	147 (77.4)	128 (79.0)	19 (67.9)		33 (86.8)	114 (75.0)	
Biologic	29 (15.3)	22 (13.6)	7 (25.0)		4 (10.5)	25 (16.5)	
Both synthetic and biologic	1 (0.5)	1 (0.6)	0 (0.0)		0 (0.0)	1 (0.7)	
No mesh used	13 (6.8)	11 (6.8)	2 (7.1)		1 (2.6)	12 (7.9)	
*Procedure type*				0.005*			0.352
Open	182 (95.8)	158 (97.5)	24 (85.7)		38 (100.0)	144 (94.7)	
Laparoscopic	7 (3.7)	3 (1.9)	4 (14.3)		0 (0.0)	7 (4.6)	
Converted to open	1 (0.5)	1 (0.6)	0 (0.0)		0 (0.0)	1 (0.7)	
Component separation performed"	50 (26.3)	41 (25.6)	9 (32.1)	0.472	10 (27.0)	40 (26.5)	0.947
Fascial defect completely closed"	173 (91.1)	147 (93.6)	26 (92.9)	0.878	35 (94.6)	138 (93.2)	0.765

Categorical variables are reported as *n* (%). Continuous variables are reported as mean ± standard deviation

*BMI* body mass index

*p < 0.05 was defined as statistically significant

"Indicates not 100% of the data was available for this variable

### Deep learning models for recurrence

**Fig. 1:**
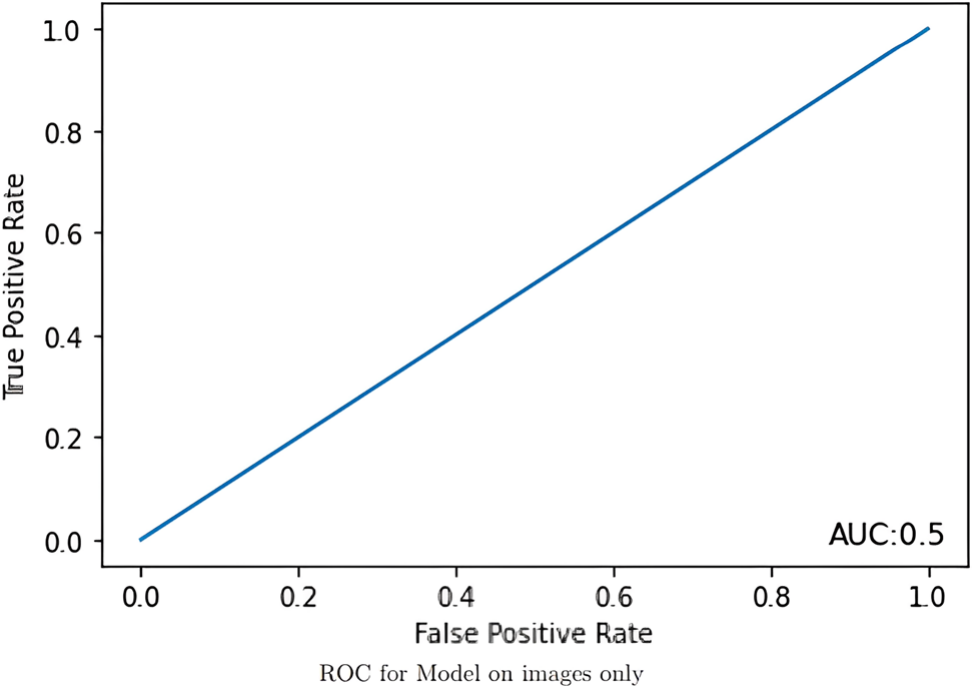
The receiver operating characteristic curve for the image-only model is shown in this figure with the area under the curve demonstrating that the model was unable to discriminate between patients who had a recurrence or not

**Fig. 2:**
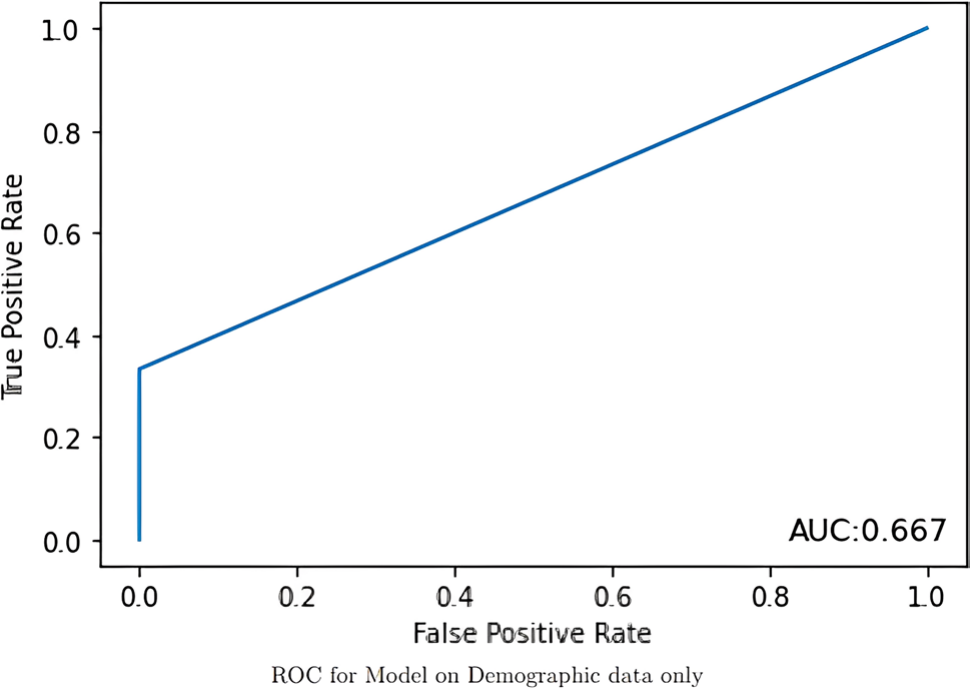
This figure shows the receiver operating characteristic curve for the clinical-only model that was more predictive of recurrence than the image-only model

**Fig. 3:**
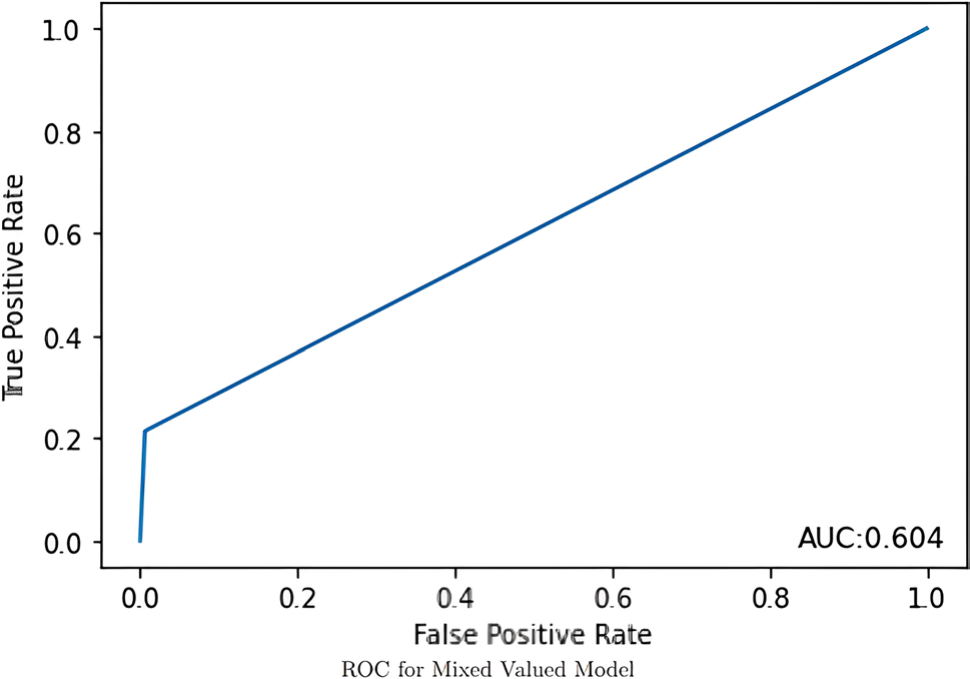
The mixed-value model incorporated the predictions of both the image-only and clinical-only models, performing better than the image-only model but not as well as the clinical-only model

The image-only DLM was not found to be a discriminatory model with an AUC of 0.500 (Fig. 1). The best performance for the clinical-only DLM was achieved when sex was excluded, so the other four variables – age, BMI, history of diabetes, and history of tobacco use – were included. This model had a training accuracy of 0.877 and a validation accuracy of 0.897 with an AUC of 0.667, outperforming the image-only model (Fig. 2). These models were then incorporated into the final mixed-value DLM that had a training accuracy of 0.875 and a validation accuracy of 0.897, but had a lower AUC of 0.604 (Fig. 3).

## Discussion

In this study, the DLM using preoperative CT images only was not predictive of hernia recurrence after AWR. The AUC of the image-only model indicated that the model was equally successful as randomly guessing. This result differed quite a bit from the prior successes of image-based DLMs in predicting other outcomes of AWR, including component separation, surgical site infection, pulmonary failure, and mesh infection [17, 18]. The hypothesis that incorporating basic clinical data into the model would improve the predictive ability of the image-based model was correct, but the final mixed-value DLM was not found to be very predictive of recurrence either. Most interestingly, the model that performed best was the DLM that utilized clinical data alone. Although this would not be considered a discriminatory model based on traditional standards, the results were nonetheless impressive given the limited amount of clinical data on which the model was given to train. In fact, the clinical-only DLM was intended only to enhance the image-based DLM and was not expected to supersede it. These findings are valuable additions to the burgeoning field of AI in the prediction of surgical outcomes. The field is still in its infancy, and it has been stressed in the literature that these models must be rigorously analyzed prior to clinical implementation [16]. Results such as these help to guide the way forward without being overly optimistic about the application of AI in augmenting surgical decision-making.

Much of the previous work with image-based DLMs to predict AWR outcomes was constructed on the rationale that specifically defined features from preoperative CT scans could be indicative of certain outcomes. For example, hernia defect size and abdominal wall thickness, measured by CT, have previously been shown to predict the need for component separation and postoperative wound complications [25]. Taking this one step further, studies by Schlosser et al. used CT volumetric analysis to predict component separation, wound complications, and respiratory insufficiency [26, 27]. Similarly, preoperative CT measurements have been shown to be predictive of achieving fascial closure during AWR [28, 29]. Some of the main drawbacks to these methods are that obtaining these measurements can be subjective, user-dependent, and time-intensive, and they typically involve specialized software and clinical expertise to define. Therefore, they do not overcome the concern of being overly cumbersome that is common to other risk stratification tools, and may be more cumbersome. This has been the purported strength of image-based DLMs: the computer learns to extract the imaging features on its own to simplify the process for clinicians. It can be postulated that previous successful DLMs have identified features from imaging that are similar to those already identified in the literature, but this has not been demonstrated. Extracting and interpreting the complex associations built by a DLM remains an extremely challenging task and is an active area of research [30].

There are a few possible reasons that the image-only DLM performed poorly in predicting hernia recurrence. Within the line of thinking that the computer is indeed “seeing” similar CT features to those that have already been studied, such as hernia dimensions, it should be acknowledged that the relationship of these features to hernia recurrence have not been well-demonstrated. In a previous study, Ballem et al. did show that a larger defect size was a risk factor for recurrence, but this finding has not held true in other studies, and a more recent meta-analysis did not show hernia dimensions to be independently predictive of recurrence [14, 31]. Additionally, another characteristic that could intuitively be extracted by the image-based DLMs is the hernia location. In particular, European Hernia Society class M1, or subxiphoid, hernias have been shown to have lower rates of tension-free fascial closure [29, 32]. However, the meta-analysis by Parker et al. did not show hernia location to be a risk factor for recurrence either [14]. The results of the present study serve as further evidence that the associations made by image-based DLMs for AWR may be fairly straightforward, aligning with hernia characteristics that have been identified in other studies. Thus, AI may perform well to predict outcomes from preoperative imaging only when CT-defined features have already been linked to these outcomes, but in this study, it did not show the ability to identify unseen characteristics to make accurate predictions.

It is also possible there were not enough instances of recurrence on which the model could train. There were 28 patients total with a recurrence; 22 were in the training set and 6 were in the testing set. In fact, the initial image-based DLM for pulmonary failure was thought to be unsuccessful for a similar reason [17]. It had used a comparable number of 29 patients with pulmonary failure, including 23 in the training set and 6 in the testing set. Initially, this had produced an unsatisfactory AUC of 0.545. The follow-up study by Ayuso et al. improved upon this model, generating an AUC of 0.70, by several different methods [18]. First, the number of patients in the total dataset was increased from 369 to 510. There were no additional positive instances of pulmonary failure added to the dataset, only patients without this outcome. Also, the raw number of patients with pulmonary failure in the training and testing sets stayed the same. Next, the model was trained in a different way, only training on the vast majority of patients who did not have pulmonary failure and learning to identify the patients who were abnormal, a strategy known as anomaly detection. At face value, this methodology would be very helpful for improving the current image-based DLM for recurrence. The main challenge is that while pulmonary failure is an outcome studied in the short term following AWR, hernia recurrence is a long-term outcome. It has been estimated that approaching the actual recurrence rate of incisional hernia repairs requires at least 10 years of follow up, a mark that very few hernia studies have achieved [8]. A strength of this study was the relatively long-term follow up, but excluding patients with shorter-term follow up also limits the ability of collecting a larger group of negative instances for the model to train on, as was done to improve the pulmonary failure model. In other words, including many patients who had short-term follow up and were considered as negative for recurrence, but who may have gone on to develop a recurrence later on, would likely introduce further variability and confusion to the model and limit its applicability to the clinical setting. A future direction for this work could be to establish a multicenter dataset that would be able to overcome these challenges, adding enough patients with preoperative imaging and long-term follow up to build a more robust image-based DLM for recurrence.

Perhaps the most unanticipated finding from this study was the predictive ability of the clinical-only DLM. In the end, the model only used four pieces of information – age, BMI, history of diabetes, and history of tobacco use – to predict hernia recurrence and far outperformed the image-only DLM as well as the mixed-value DLM. Notably, all four of these demographics have been shown to be risk factors for recurrence on meta-analysis [14]. The surprising part is that there was no statistical difference between these variables in patients with or without a recurrence in the present study. With this sample size it is also possible that this finding may represent a type II error, but this observation could highlight the ability of DLMs to find complex associations between input variables that standard statistical methods fail to identify [16]. Another recent study by Hassan et al. also used AI, analyzing clinical data only, to predict recurrence as well as surgical site occurrences and 30-day readmissions [19]. Their models outperformed traditional multivariable logistic regression models in predicting these outcomes. Furthermore, Holihan et al. described the flaws in several predictive models they created for ventral hernia recurrence that were built with regression analysis, showing that they all performed poorly on external validation [33]. The unexpected success of the clinical-only DLM in this study further establishes the advantages of AI over conventional statistical models that may fail to identify the complex, nonlinear associations between data.

To summarize, this study reports on the comparative success of three different DLMs in predicting hernia recurrence following AWR. While all three models were poorly predictive of recurrence, the clinical-only DLM was the most predictive. The image-only DLM in this study showed no ability to discriminate between patients who would or would not develop a recurrence based on their preoperative CT imaging. A mixed-value DLM incorporating image data and clinical data also performed poorly. In the context of multiple successful image-based DLMs predicting AWR outcomes that have been published previously, these results may reflect the limitations of image-based deep learning for predicting recurrence specifically or, more generally, the difficulties and complexities of accurately studying this outcome in AWR. On the other hand, the predictive ability of the DLM using only very few clinical data was encouraging, and building on this model with additional demographic information is a worthy direction for future work.
